# Micro-RNA 92a as a Therapeutic Target for Cardiac Microvascular Dysfunction in Diabetes

**DOI:** 10.3390/biomedicines10010058

**Published:** 2021-12-28

**Authors:** Mostafa Samak, Diana Kaltenborn, Andreas Kues, Ferdinand Le Noble, Rabea Hinkel, Giulia Germena

**Affiliations:** 1 Laboratory Animal Science Unit, Leibnitz-Institut für Primatenforschung, Deutsches Primatenzentrum GmbH, Kellnerweg 4, 37077 Göttingen, Germany; MSamak@dpz.eu (M.S.); DKaltenborn@dpz.eu (D.K.); AKues@dpz.eu (A.K.); ggermena@dpz.eu (G.G.); 2 DZHK (German Centre for Cardiovascular Research), Partner Site Göttingen, 37075 Göttingen, Germany; 3 Department Cell and Developmental Biology, Karlsruhe Institute of Technology, Fritz Haber Weg 4, 76131 Karlsruhe, Germany; ferdinand.noble@kit.edu; 4 Institute of Experimental Cardiology, University of Heidelberg, 69120 Heidelberg, Germany; 5 DZHK (German Centre for Cardiovascular Research), Partner Site Heidelberg/Mannheim, 69120 Heidelberg, Germany; 6 Institute for Animal Hygiene, Animal Welfare and Farm Animal Behaviour (ITTN), Stiftung Tierärztliche Hochschule Hannover, University of Veterinary Medicine, 30173 Hannover, Germany

**Keywords:** endothelial function, inflammation, angiogenesis, mir-92a, diabetes

## Abstract

Microvascular dysfunction is a pathological hallmark of diabetes, and is central to the ethology of diabetes-associated cardiac events. Herein, previous studies have highlighted the role of the vasoactive micro-RNA 92a (miR-92a) in small, as well as large, animal models. In this study, we explore the effects of miR-92a on mouse and human cardiac microvascular endothelial cells (MCMEC, HCMEC), and its underlying molecular mechanisms. Diabetic HCMEC displayed impaired angiogenesis and a pronounced inflammatory phenotype. Quantitative PCR (qPCR) showed an upregulation of miR-92a in primary diabetic HCMEC. Downregulation of miR-92a by antagomir transfection in diabetic HCMEC rescued angiogenesis and ameliorated diabetic endothelial bed inflammation. Furthermore, additional analysis of potential in silico-identified miR-92a targets in diabetic HCMEC revealed the miR-92a dependent downregulation of an essential metalloprotease, ADAM10. Accordingly, downregulation of ADAM10 impaired angiogenesis and wound healing in MCMEC. In myocardial tissue slices from diabetic pigs, ADAM10 dysregulation in micro- and macro-vasculature could be shown. Altogether, our data demonstrate the role of miR-92a in cardiac microvascular dysfunction and inflammation in diabetes. Moreover, we describe for the first time the metalloprotease ADAM10 as a novel miR-92a target, mediating its anti-angiogenic effect.

## 1. Introduction

Diabetes mellitus (DM) is an established risk factor for cardiovascular disease (CVD) [1]. In this regard, diabetic patients exhibit a cardiovascular phenotype characterized by capillary rarefication, pericyte loss and impaired coronary perfusion [2]. DM-induced macro- and microvascular dysfunction predisposes patients to exacerbated myocardial tissue damage upon ischemic incidents [3]. Moreover, diabetic hearts typify a state of contractile dysfunction and ventricular stiffness, characterized by hypertrophy and apoptosis of cardiac myocytes, i.e., diabetic cardiomyopathy [4]. Importantly, vascular inflammation is another pathological hallmark of diabetes leading to increased risk of atherosclerosis [5].

Previously published work by Hinkel et al. also demonstrated the beneficial effects of targeting specific microRNAs (miRs), i.e., miR-92a, in a porcine model of myocardial ischemia [6]. Furthermore, in their studies miR-92a was one out of three miRNAs that were significantly up-regulated in the porcine diabetic myocardium, even without cardiovascular events [2]. Nevertheless, the role of miR-92a in diabetic microvascular dysfunction and/or cardiomyopathy remains unclear.

On a molecular level, our previous data (unpublished) from Affymetrix chip analysis in human endothelial cells, as well as in silico research of miR-92a targets revealed several possible indirect as well as direct targets, some of which are key players in vascular biology. Among these targets, A disintegrin and metalloproteinase 10 (ADAM10) is a member of a large family of endopeptidases with a sheddase activity involved in activation of ligand-induced Notch receptor, a key regulator of angiogenesis, and was recently shown to control the coronary arterial endothelium [7,8]. ADAM10 is a predicted target of miR-92a, by TargetScan database with a conserved seed-sequence match in both mouse and human. However, the role of ADAM10 in diabetic endothelial dysfunction has not been previously explored.

Several players of diabetic vascular inflammation are predicted targets of miR-92a. These include the Krüpple-like factors KLF2 and KLF4 [9,10]. KLF2 and KLF4 are well characterized transcription regulators of several anti-inflammatory, anti-oxidant and anti-thrombotic genes; they exert crucial vascular protective roles, deficient in pathologic conditions [11,12]. It is, however, so far unclear whether miR-92a regulates these factors in the context of diabetic vascular inflammation.

In this study, we aimed to characterize endothelial dysfunction in human cardiac microvascular endothelial cells (HCMEC) from diabetic patients, translate the previous findings on miR-92a in porcine models, and elucidate its underlying molecular mechanism by addressing the aforementioned downstream targets in both human and mouse in vitro systems.

## 2. Materials and Methods

### 2.1. EC Culture

Primary ventricular HCMEC (PromoCell C-12286) obtained from a 38-year-old Caucasian female (Lot #446Z001.1) or 63-year-old non-diabetic (Lot no. 447Z026.3) or type-II diabetic (Lot no. 451Z015.1). Caucasian males were cultured in PromoCell microvascular media MV (C-22020) or MV2 (C-22022) media, supplemented with their corresponding supplement mixes (C-39225 or C-39226, respectively) and 0.1% penicillin/streptomycin (PS). Cells were kept in a humidified incubator at 37 °C Celsius and 5% CO_2_, and used for experiments between passages 2 and 8.

Mouse cardiac microvascular endothelial cells (MCMEC) from Cedarlane (CLU510) were cultured according to provider in DMEM with 10 mM HEPES, 1% PS and 5% fetal bovine serum (FBS).

THP-1 monocytes (ATCC TIB-202) were maintained in RPMI medium with 10% FBS, 0.05 mM 2-mercaptoethanol and 1% PS.

### 2.2. Transfection

Cells in culture were transfected at 80% confluence in MV2 (HCMEC) or serum-free DMEM (MCMEC) with Lipofectamine RNAiMAX reagent (Invitrogen—cat. no. 13778150) and small RNA at a concentration of 10 nM using manufacturer’s protocol. Transfection complexes were prepared in OptiMEM medium, added and incubated with the cells for 4–6 h, followed by medium change and incubation for additional 24 h in complex-free medium before cells were used for angiogenesis assays. For all other assays, the cells are collected by trypsinization directly after the following small RNA (Ambion) were used in this study: Anti-miR™ hsa-miR-92a-3p (Ant-92a) (AM10916); anti-miR™ Negative Control (Ant-Ctrl) (AM17010); pre-miR™ miRNA precursors hsa-miR-92a (PM10916) and mmu-miR-92a (PM10312) (Pre-92a); pre-miR™ miRNA precursor negative controls (Pre-Ctrl) (AM17110/AM17111); Silencer^®^ siRNA ADAM10 (4390824 IDs1006) (siADAM10); Silencer^®^ siRNA Adam10 (AM16708 ID59937) (siAdam10); Silencer^®^ siRNA negative controls (4390844/4390846).

### 2.3. Tube Formation

**Figure 1:**
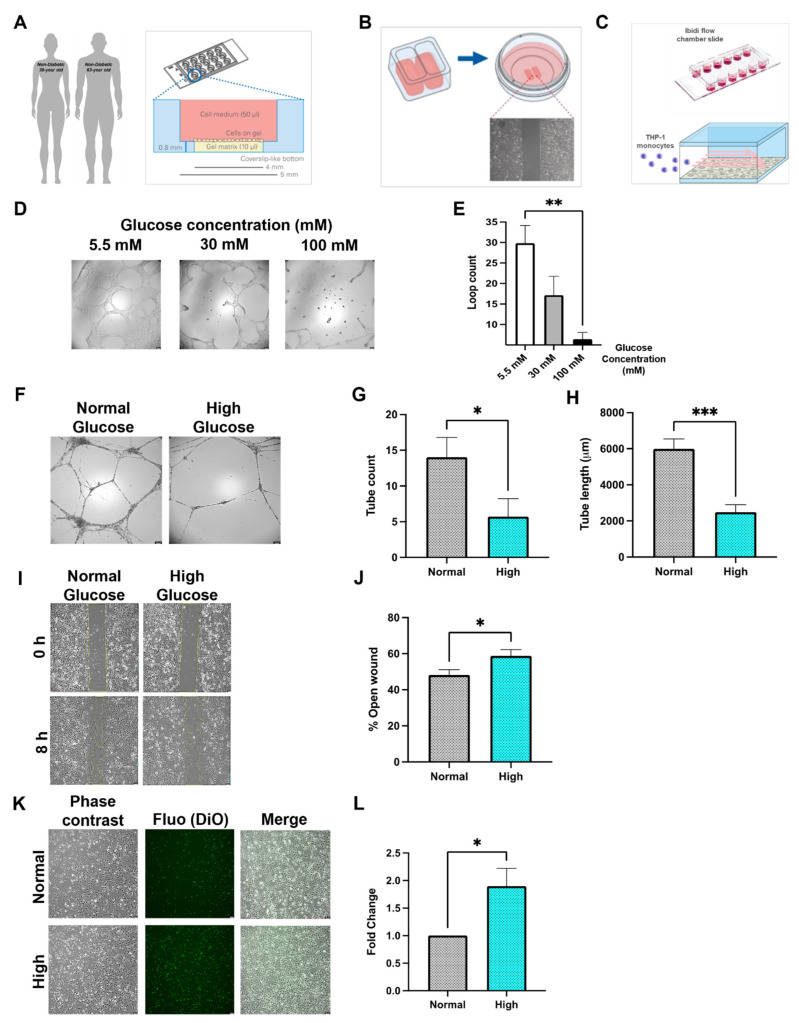
Characterization of MCMEC and HCMEC behaviour in normal and high glucose Schematic representation of the angiogenesis (**A**), wound healing (**B**) and flow chamber (**C**) techniques performed with Ibidi chambers. (**D**,**E**) Tube formation of MCMEC on Matrigel at different glucose concentration (mM). Bright field images and analysis of total loop count. (**F**–**H**) Tube formation of HCMEC on Matrigel, phase contrast images and analysis of tube formation parameters of HCMEC on Matrigel in normal (5.5 mM) or high (30 mM) glucose. (**I**,**J**) Examples and quantification of wound area after 8 h in normal and high glucose. (**K**,**L**) Phase contrast, fluorescent images and analysis (fold change) of adherent THP-1 (DiO-labeled) count on HCMEC monolayer in flow chamber assay under normal and high glucose. Statistical analyses by student *t*-test or by one-way ANOVA (*n* ≥ 4). Scale bars = 100 μM. * *p* < 0.05; ** *p* < 0.01; *** *p* < 0.001.

Angiogenesis by tube formation was performed in μ-Slide Angiogenesis (Ibidi 81506) (Application Note A19). A total of 10 μL Matrigel^®^ matrix basement membrane (Corning^®^ 354234) was pipetted in the slide inner-wells, and an average of 12,500–13,000/well endothelial cells were seeded on top (outer-well). Tube formation was examined after 12 h of incubation and bright-field 5×-microscopic images were taken by Leica DMi8 (Leica Microsystems, Wetzlar, Germany) (Figure 1A).

### 2.4. Wound Healing

Wound healing assay was performed in a Culture-Insert 2 Well in μ-Dish 35 mm (Ibidi 80206). Cell suspensions of 5 × 10^5^ cells/mL were prepared and 70 μL were added in each well, and cells were incubated for 24–36 h to form monolayers. The inserts were then carefully removed and the cells were washed with calcium and magnesium-free phosphate buffer saline (PBS), and fresh medium was added. Phase contrast 5×-microscopic images were taken at time intervals 0, 8 and 24 h (Figure 1B).

### 2.5. Flow Chamber Assay

HCMEC suspensions were prepared at a 5 × 10^5^ cells/mL, and 40 μL were added to each channel of μ-Slide VI^0.4^ (Ibidi 80606). Cells were incubated for 24–36 h to form stable monolayers. The flow chamber experiment was adapted from Stachel et al., 2013 [13]. Briefly, THP-1 monocytes were stained with Vybrant^TM^ Cell-Labeling Solutions DiI (V-22885) or DiO (V-22886) according to manufacturer’s protocol. Cells were washed with PBS and a concentration of 7.5 × 10^5^ cells/mL was prepared in their culture medium. A perfusion system was established; two 50 mL Original-Perfusor^®^ syringes (Braun) were filled with THP-1 cell suspension or MV2 medium (washing medium), connected with perfusion lines to a 3-way valve and placed each in Perfusor^®^ Space pumps (Braun). A tube adapter set (Ibidi 10831) was connected to the valve and in the flow chamber inlets. A flow of 47.4 mL/hour was established to simulate venous shear stress 1 dyn/cm^2^ and flow round was run for each channel as the follows: 2 min washing medium, 5 min cell suspension and 2 min washing medium. The flow slide was then imaged by phase contrast and fluorescent imaging (Leica Microsystems, Germany) (Figure 1C).

### 2.6. Proliferation Assay

A total of 10,000 cells were seeded in fibronectin-coated (Sigma-Aldrich F1141, Burlington, MA, USA) μ-Slide 8 Well chambered coverslip (Ibidi 80806) and incubated overnight. Proliferation assay was performed following the manufacturing instructions of the Click-iT™ Plus EdU Cell Proliferation Kit (Alexa Fluor™ 488) (Invitrogen C10637). Briefly, cells were treated with 10 μM EdU for 24 h, fixed with 3.7% formaldehyde, and imaged with fluorescent microscopy (Leica Microsystems, Germany). Proliferating cells were detected by positive EdU staining. The percentage of proliferating cells was calculated on the total cell number quantified by Hoechst staining.

### 2.7. Western Blot

Cells were collected by trypsinization, centrifuged at 250 RCF for 5 min and washed with calcium and magnesium-free PBS and centrifuged at 250 RCF for 5 min. Cells were lysed for 10 min on ice in lysis buffer (10 mM Tris/HCl pH 7.5; 150 mM NaCl; 0.5 mM EDTA; 0.5% NP-40) containing proteases and phosphatases inhibitor cocktail (Roche). After clarification, protein concentration was quantified and the different samples were resuspended in sample buffer. Western blots were performed and immunoblots were incubated with the following antibodies: anti-GAPDH, anti-ADAM10 (Abcam) at a 1:1000 dilution.

### 2.8. Quantitative PCR

Cells were collected by trypsinization, centrifuged at 250 RCF for 5 min and small and/or large RNA was extracted by NucleoSpin^®^ miRNA kit (Macherey & Nagel, Dueren, Germany). For gene expression analysis, 500 ng RNA were used for cDNA synthesis by Omniscript^®^ Reverse Transcription kit (Qiagen 205113). Quantitative PCR was run using TaqMan™ Fast Advanced Master Mix (Applied Biosystems 4444557, Waltham, MA, USA) and the following TaqMan assays (primers): human beta-actin (ACTB) (Hs99999903_m1); ADAM10 (Hs00153853_m1); KLF2 (Hs00360439_g1); KLF4 (Hs00358836_m1); mouse beta-actin (Actb) (Mm02619580_g1); Adam10 (Mm00545742_m1).

For miRNA quantification, 20 ng RNA were used for the cDNA synthesis using TaqMan™ Advanced miRNA cDNA Synthesis Kit (A28007) and TaqMan™ Advanced miRNA Assay (A25576) and the following assays for both human and mouse: hsa-miR-92a-3p (assay ID 477827_mir) and hsa-miR-26a-5p (assay ID 477995_mir) as endogenous control. Real-Time PCR (RT-PCR) was run using the recommended thermal cycling profiles and StepOnePlus™ software v2.3 (Applied Biosystems) to calculate the comparative C_T_ (relative quantification).

### 2.9. ImageJ Analysis

Tube formation was analyzed using Angiogenesis Analyzer software of ImageJ [14]. A tube in our analysis is the structure referred to by the program as “master segment”, and was included in measures if it contributed to a continuous mesh structure. To minimize artifacts, a threshold of 100–200 pixels was set for the master segment size. Two parameters were included as endpoints for tube formation measures, total tube length in micrometers and tube count.

Wound healing was analyzed using ImageJ polygon selection to trace and measure the open wound area on native phase contrast images. Wound healing is represented as percentage of open wound at a later time point relative to the initial wound area upon chamber removal (time 0).

Adherent THP-1 to endothelial monolayers in flow chambers were counted by particle number quantification in ImageJ. Briefly, images were split in 3 color components, and depending on the dye (DiI or DiO), the red- or green-colored image was subjected to threshold adjustment. Particle analysis settings were applied and particles were counted.

### 2.10. Immunofluorescence

Myocardial tissue from *INS*^C94Y^ transgenic diabetic pigs and non-diabetic littermates were obtained from Institute of Molecular Animal Breeding and Biotechnology, Gene Center, LMU Munich [2]. Cryosections were stained for ADAM10 (Abcam) and PECAM-1 (Thermo Fischer, Waltham, MA, USA), 1:100 dilution, followed by Alexa-488 and Alexa-594 secondary antibody (Thermo Fischer). Forty times magnification images were taken by fluorescence microscopy (Leica Microsystems, Germany) and analyzed by its software system.

Endothelial tube networks were fixed with 4% paraformaldehyde, washed with PBS and blocked for one hour with 5% bovine serum albumin (BSA). A total of 20 μg/mL rabbit anti-ADAM10 antibody (Invitrogen) was prepared in 5% BSA and incubated with the cells for 8 h, washed with PBS, incubated with Alexa Fluor-488-coupled secondary goat anti-rabbit antibody (Invitrogen) for 2 h and finally washed with PBS. The 10× or 20× images were taken by fluorescence microscopy (Leica Microsystems, Germany).

### 2.11. Statistical Analysis

Data were analyzed using GraphPad Prism software and were presented as mean ± SEM (error bars). Sample size and experimental replicates were indicated in figure legends. Statistical analysis was performed by Student’s t test or one-way ANOVA. *p* values, * *p* < 0.05; ** *p* < 0.01; *** *p* < 0.001; and **** *p* < 0.0001 were considered statistically significant.

## 3. Results

### 3.1. Diabetes In Vitro and Its Relevance for Microcirculatory EC Function

Analysis of angiogenic potential of MCMEC in different glucose concentrations revealed a reduced loop formation upon increasing glucose levels (Figure 1D,E). MCMEC normally request 30 mM of glucose in their culture medium; therefore, we further increased the glucose concentration in their medium to 100 mM to achieve a relatively high glucose situation. Since 100 mM of glucose is a very high experimental value, which does not reflect the diabetic situation in human, we decided to use primary human EC of cardiac microcirculatory origin, i.e., HCMEC.

Analyzing the angiogenic potential of HCMEC cultured in relevant high-glucose (30 mM) revealed a significant reduction in tube formation parameters such as total tube length and count compared to those in normal-glucose (5.5 mM) (Figure 1F–H). Furthermore, the migratory capacity of the HCMEC in high-glucose was clearly reduced compared to those in normal-glucose (Figure 1I,J). The inflammatory response of HCMEC to high-glucose in vitro was demonstrated in a flow-chamber assay, where adhesion of THP-1 cells was significantly increased compared to normal-glucose (Figure 1K,L).

Interestingly, qPCR analysis of miR-92a in non-diabetic HCMEC cultured in high glucose showed a significant upregulation (Figure S1A).

**Figure 2:**
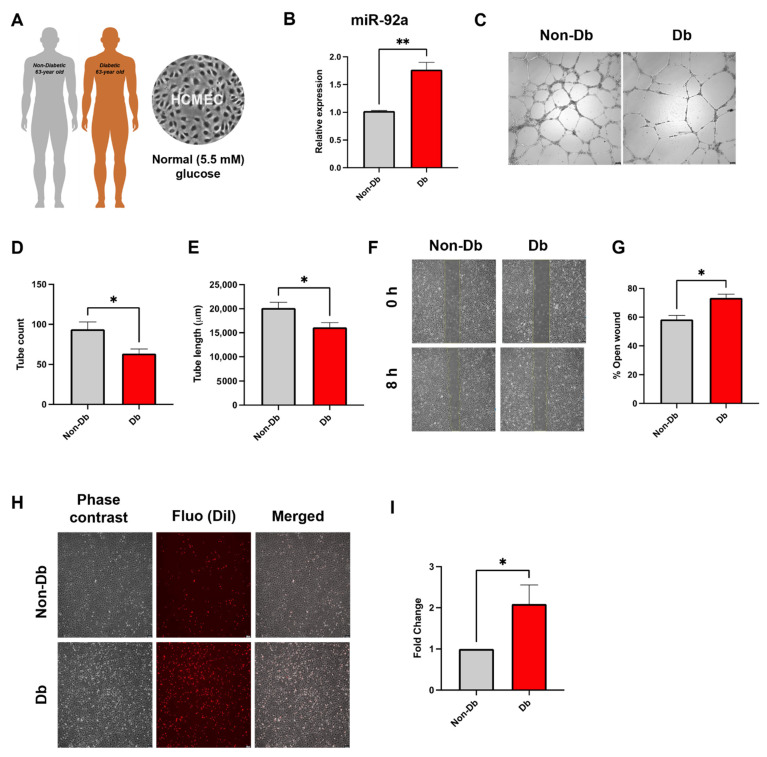
Characterization of type-II diabetic HCMEC. (**A**) Primary HCMEC from 63-year-old caucasian type-II diabetic (Db) patient or age-matched non-diabetic (Non-Db) cultured in normal (5.5 mM) glucose. (**B**) qPCR of miR-92a. (**C**–**E**) Tube formation of HCMEC on Matrigel. Bright field images and analysis of total tube length and tube count. (**F**,**G**) Phase contrast images and analysis of relative wound area at 8 h. (**H**,**I**) Phase contrast, fluorescence images and merge as well as analysis of adherent THP-1 (DiI-labeled) count (fold change) on HCMEC monolayer. Statistical analyses by Student’s *t*-test (*n* = 4). Scale bars = 100 μM. * *p* < 0.05; ** *p* < 0.01.

To verify the previous findings in a pathologically relevant model, HCMEC from a diabetic donor were used for the following experiments (Figure 2A). We first analyzed miR-92a expression in diabetic HCMEC, since this was one of the significantly up-regulated miRNAs in the diabetic pig heart tissue. Quantitative PCR analysis showed significantly elevated levels of miR-92a in diabetic HCMEC compared to matched non-diabetic controls (Figure 2B).

Characterization of the angiogenic potential of type-II diabetic HCMEC revealed significant reduction in their ability to form tubes when cultured on extracellular matrix gel (Matrigel) compared to age-matched non-diabetic controls as evident from quantification of total tube length as well as tube count (Figure 2C–E). Furthermore, diabetic HCMEC showed a significantly compromised wound healing ability, manifested in relatively larger wound areas compared to matched non-diabetic controls (Figure 2F,G).

### 3.2. Inflammatory Phenotype of Diabetic HCMEC

Endothelial beds from diabetic HCMEC displayed a pronounced inflammatory character in flow-chamber assays evident in their significantly increased retention of monocytes compared to those from matched non-diabetic controls (Figure 2H,I).

### 3.3. Downregulation of miR-92a Reverses Diabetic Phenotype

**Figure 3:**
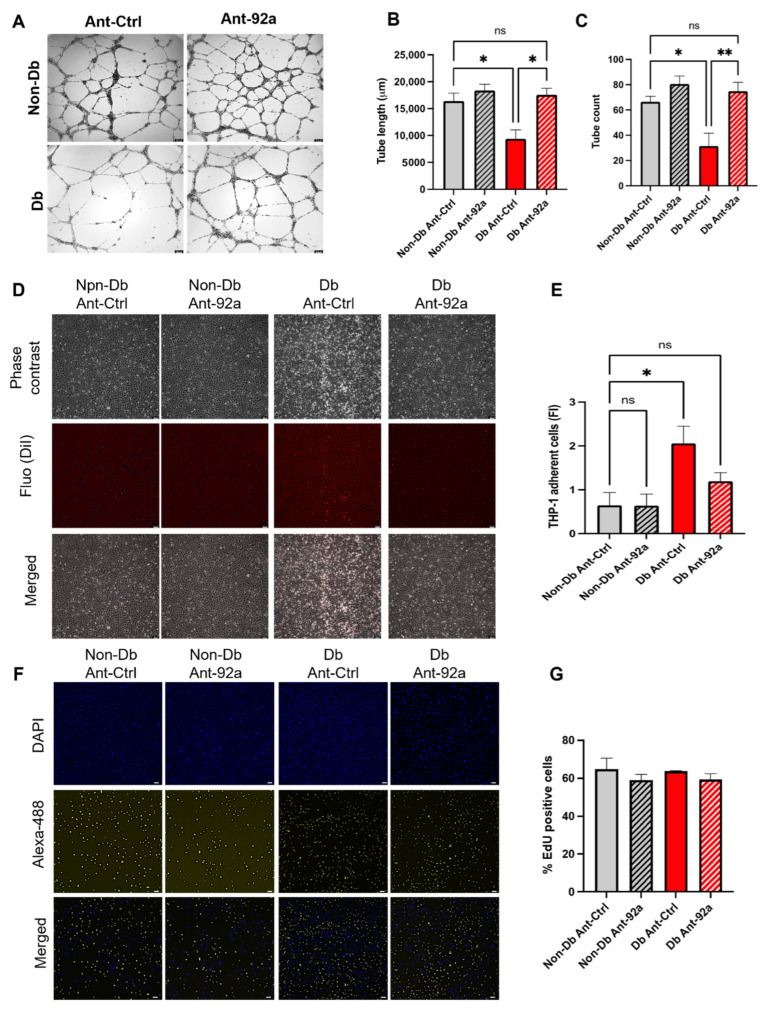
Inhibition of miR-92a rescues diabetic phenotype in HCMEC. (**A**–**C**) Bright field images and analysis of tube formation parameteres in Db or Non-Db HCMEC upon downregulation of miR-92a (Ant-92a) or control (Ant-Ctrl). (**D**,**E**) Phase contrast images, fluorescence images and analysis (fold change) of adherent THP-1 (DiI-labeled) count on Db or Non-Db HCMEC monolayer upon downregulation of miR-92a (Ant-92a) or control (Ant-Ctrl). (**F**,**G**) Fluorescence images and analysis of cell proliferation by EdU incorporation in primary Db or Non-Db HCMEC upon downregulation of miR-92a (Ant-92a) or control (Ant-Ctrl). Statistical analyses by one-way ANOVA (*n* ≥ 3). Scale bars = 100 μM. ns = not significant; * *p* < 0.05; ** *p* < 0.01.

Since miR-92a was shown to be one of the most upregulated miRNAs in endothelial cells (Figure 2B) and in pig cardiac tissue [2], we investigated in a next step the inhibition of miR-92a by antagomir transfection. Here, down-regulation of miR-92a (Figure S1B) significantly enhanced angiogenic potential of diabetic HCMEC manifested in increased total tube length and tube count compared to control-transfected cells (Figure 3A–C). In addition, ablation of miR-92a in diabetic HCMEC rescued the observed inflammatory phenotype by reducing monocyte adhesion to endothelial monolayer compared to control transfected cells (Figure 3D,E).

Interestingly, miR-92a inhibition did not influence proliferation of HCMEC from either diabetic or non-diabetic patients as measured by EdU-incorporation assay (Figure 3F,G).

### 3.4. MiR-92a Downstream Targets

**Figure 4:**
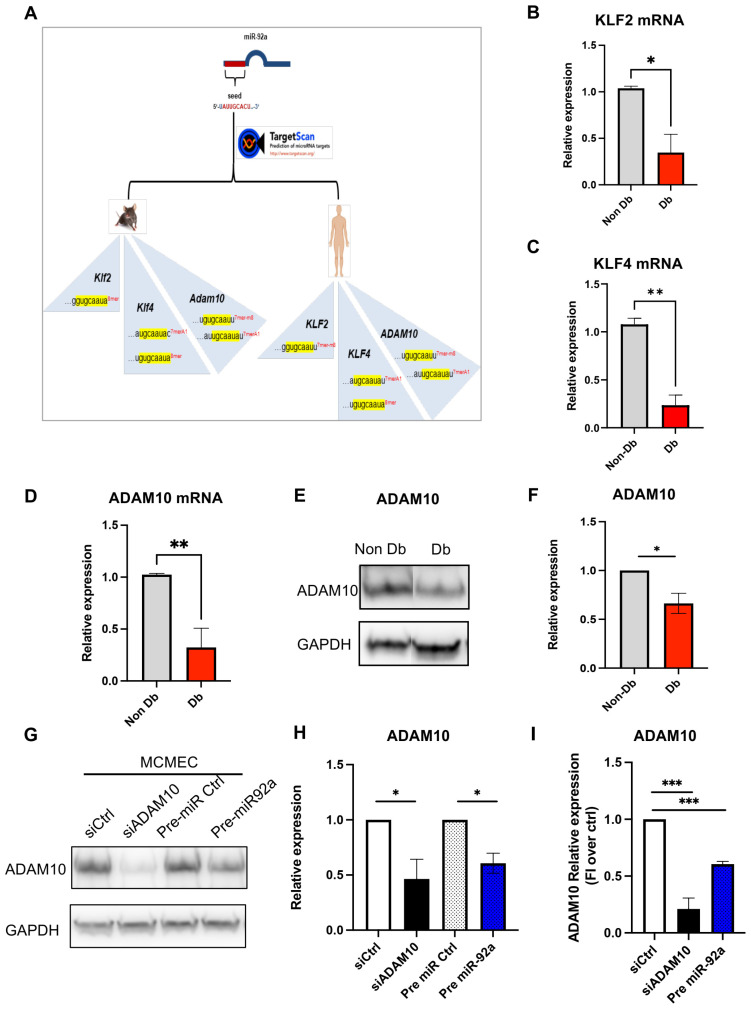
miR-92a downstream targets. (**A**) TargetScan in silico prediction of conserved downstream targets of miR-92a. (**B**–**D**) qPCR of KLF2 (**B**), KLF4 (**C**) and ADAM10 (**D**) in Db or Non-Db HCMEC. (**E**,**F**) Western blot analysis and relative quantification of ADAM10 in Db or Non-Db HCMEC. (**G**,**H**) Western blot analysis and relative quantification of ADAM10 upon overexpression of miR-92a (Pre-92a) compared to siRNA-mediated downregulation of Adam10 (siAdam10) in MCMEC. (**I**) Relative quantification of ADAM10 upon overexpression of miR-92a (Pre-92a) compared to siRNA-mediated downregulation of Adam10 (siAdam10) in HCMEC. Statistical analysis by unpaired *t*-test or one-way ANOVA (*n* = 3). * *p* < 0.05; ** *p* < 0.01; *** *p* < 0.001.

In silico analysis of miR-92a downstream targets by TargetScan prediction revealed several conserved targets bearing miR-92a seed sequence matches in their 3′-UTR. In the present study, we confined our selection to three conserved targets, i.e., ADAM10, KLF2 and KLF4, with crucial functions in EC tube formation (angiogenesis), migration and inflammation. Dysregulated levels of these targets downstream of miR-92a can explain the observed diabetic microvascular phenotype. The conserved seed sequence of miR-92a 3-*p* is 5′-UAUUGCACU-3′, and it was found to target mRNA sequences of the selected targets in human and mouse (Table 1 and Figure 4A):

Congruent with previous finding of elevated miR-92a expression in diabetic HCMEC, qPCR of its predicted targets *ADAM10*, *KLF2* and *KLF4* revealed significant reduction in their expression levels in diabetic HCMEC compared to matched non-diabetic control (Figure 4B–D). On protein level, Western blot analysis of ADAM10 showed significant reduction in protein lysates from diabetic HCMEC compared to matched non-diabetic controls (Figure 4E,F).

Accordingly, over expression of miR-92a by pre-miR transfection (Figure S1C), quantification shown in supplementary Figure S1C, in MCMEC led to downregulation of *Adam10* gene expression and ADAM10 protein levels by qPCR and Western blot, respectively (Figure 4G,H). Similarly, over expression of miR-92a led to downregulation of ADAM10 protein levels in non-diabetic HCMEC (Figure 4I and Figure S1D).

### 3.5. ADAM10 Is Important for Microvascular Endothelial Behavior

**Figure 5:**
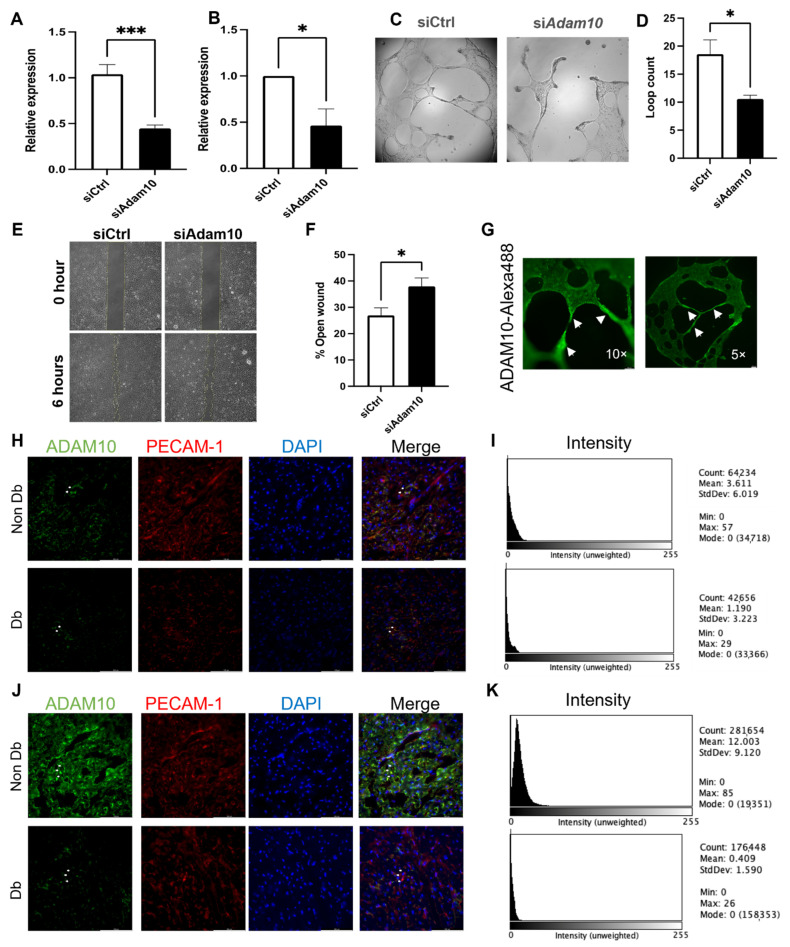
ADAM10 is important for microvascular endothelial behavior. (**A**) qPCR and (**B**) Western blot quantification of ADAM10 upon ADAM10 siRNA overexpression in MCMEC. (**C**,**D**) Bright field images and analysis of MCMEC angiogenesis (loop count) upon downregulation of Adam10 by targeted siRNA compared to control. (**E**,**F**) Phase contrast images and analysis of wound area at in MCMEC upon downregulation of Adam10 in 6 h wound healing assay. (**G**) Immunocytochemistry of ADAM10 (green) localization (white arrows) during tube formation in MCMEC. (**H**–**K**) Immunohistochemistry of ADAM10 (green), PECAM-1 (red) and nuclei (DAPI) in micro- and macrocirculation of Db or Non-Db pig heart slices. Co-localisation marked in merged images with white arrows. (**I**,**K**) Fluorescence intensity measurment of ADAM10 expression of corresponding images. Statistical analysis by Student’s *t*-test (*n* = 4). Scale bars = 100 μm. * *p* < 0.05; *** *p* < 0.001.

Ablation of ADAM10 in MCMEC by transfection of siRNA against *Adam10* mRNA significantly inhibited angiogenesis on Matrigel evident in reduced loop count compared to control transfected cells (Figure 5A–D). Additionally, downregulation of ADAM10 in MCMEC by targeted siRNA transfection significantly impaired wound healing compared to control transfected cells (Figure 5E,F). Furthermore, ADAM10 staining in tube formation assay shows an increased ADAM10 signal in the endothelial cell areas with high angiogenic activity (Figure 5G). In pig heart slices of diabetic and non-diabetic pigs the PECAM-1/ADAM10 staining showed a reduced ADAM10 intensity in macro- and microcirculation (Figure 5H–K). Taken together, these show a clear impairment of angiogenic abilities of endothelial cells upon diabetic condition. Furthermore, these data suggest that miR-92a via ADAM10 regulation is partly responsible for the impaired angiogenic phenotype in endothelial cells.

## 4. Discussion

Vascular complications are the main culprit for diabetes-associated morbidities and mortalities [15]. Impaired vascularization predisposes the diabetic myocardium to ischemic injury and aggravates its outcome. Both macro- and microcirculation in the heart are inflicted by diabetes, where endothelial dysfunction is a common denominator [2,16]. In this context, several micro-RNAs have been shown to be dysregulated in diabetes [17]. In fact, hyperglycemia can per se dysregulate subcellular components of micro-RNA biogenesis and processing [18]. Herein, miR-92a has been shown to be among micro-RNAs upregulated in the hearts of diabetic pig models of type I DM. miR-92a is of a particular interest as it belongs to a polycystronic cluster of vasoactive micro-RNAs, i.e., miR-17~92 [19,20]. In this study, and in congruence with previous findings in porcine models, we show for the first time an upregulation of miR-92a in cardiac microvascular endothelial cells from human patients of type II DM. This finding highlights the direct link between the state of hyperglycemia in either type of DM and upregulation of miR-92a, and corroborated by our data showing increased miR-92a expression in non-diabetic endothelial cells cultured in higher glucose. Furthermore, these findings imply a direct contribution of cardiac endothelia to the observed upregulation of miR-92a in the diabetic myocardia. It is, however, not yet known if other cells types in the diabetic myocardium also upregulate miR-92a.

Pre-clinical studies by Hinkel and colleague demonstrated therapeutic benefits in diabetic pig models of myocardial ischemia upon inhibition of miR-92a [6]. Delivery of locked-nucleic antisense (LNA) to miR-92a enhanced collateralization and prevented capillary rarefaction in these models. In line with these findings, we show here that inhibition of miR-92a enhanced tube formation (angiogenesis) of cardiac microvascular endothelial cells from human patients. Breaking down the cellular behavior of tube formation, it is primarily a function of migration and proliferation of tip and stalk cells [21,22]. Furthermore, both migration and proliferation are involved in the so called “wound healing” of EC. Interestingly, miR-92a inhibition did not influence HCMEC proliferation, suggesting that the observed angiogenic phenotype is confined to migration.

On a molecular level, in silico analysis of miR-92a targets revealed two complementary sites for its seed sequence in the 3′-UTR of *ADAM10* mRNA. ADAM10 is a metalloproteinase which has been shown to play pivotal role in vascular development [7]. Several reports have shown that ADAM10 is indispensable for the activation of Notch receptor, the primary driver of tip cell behavior in EC [23]. Importantly, the ADAM10/Notch has been recently shown to control coronary arterial specification and EGFR signaling, maintaining homeostasis in coronary endothelial bed [8]. Here, we report significantly downregulated levels of ADAM10 in diabetic HCMEC compared to non-diabetic controls, which can explain the observed diminished angiogenic parameters in those cells. We also show an inverse relationship between miR-92a and ADAM10 levels in microvascular EC; and that lack thereof compromises their angiogenic as well as wound healing potential.

Chronic vascular inflammation is a well characterized pathological observation in diabetes culminating in atherosclerosis and hemodynamic perturbations. In this regard, the diabetic endothelium responds by upregulation of inflammatory markers, including vascular adhesion molecule 1 (VCAM1), intercellular adhesion molecule 1 (ICAM1) and monocyte chemoattractant protein 1 (MCP1), among others [24,25]. In a recent study by Song et al., overexpression of these markers has been shown to increase monocyte adhesion to HUVEC under inflammatory conditions [9]. Here, we show that primary HCMEC from diabetic patients display the same inflammatory phenotype, and that inhibition of miR-92a leads to its amelioration. In the aforementioned study, Song and colleagues highlighted the importance of KLF2 in induction of transcription factor EB (TFEB) under shear stress, which dampens the inflammatory signaling and the expression of the aforementioned inflammatory markers. Importantly, they showed that TFEB activity was downregulated in diabetic mice. KLF2 is a verified miR-92a target; and in the present study we report significantly lower levels of KLF2 in diabetic HCMEC compared to non-diabetic controls. Inhibition of miR-92a in diabetic HCMEC is therefore expected to restore KLF2 levels, more likely explaining the anti-inflammatory effects of miR-92a antagomir treatment.

Similarly, KLF4 is well-recognized for its anti-inflammatory and anti-atherogenic roles [12]. Furthermore, KLF4 was characterized for its important roles in endothelial differentiation and angiogenesis by regulation of Notch signaling [26,27]. With two conserved complementary sites for miR-92a seed sequence in its mRNA 3′-UTR, we reported lower levels of KLF4 in diabetic HCMEC. We are currently investigating the direct effects of miR-92a modulation on the expression of both KLFs, and weather restoration of their levels can ameliorate the observed inflammatory phenotype in diabetic microvasculature.

Altogether, our data verifies important downstream targets of miR-92a in EC. Dysregulation of these targets in the diabetic cardiac microcirculation can indeed compromise vascular homeostasis and partially explains the underlying pathogenesis of diabetic microvascular dysfunction. Furthermore, our present data extrapolate the previous reports from animal models as well as HUVEC on miR-92a to cardiac microcirculation, more specifically from human patients. In this regard, it is important to state that the scarcity of donors represent a limitation to studies on primary cells. Nevertheless, our data calls for further research in a clinical context. For example, miR-92a might serve as a valuable prognostic biomarker for cardiovascular and/or metabolic disease. Furthermore, targeting micro-RNAs as a therapeutic intervention for cardiovascular disease has been on the rise in the recent years [28]. To this end, locked nucleic acid-based antisense oligonucleotide (LNA) therapies are promising tools. Importantly, a clinical study from last year by Abplanalp, Dimmeler et al. described MRG-110 as an effective LNA to target miR-92a in peripheral blood [29]. This, along with the present study, qualify miR-92a as a promising therapeutic target for diabetes-associated cardiac microvascular dysfunction.

## Figures and Tables

**Table 1:** Analysis results of miR-92a downstream targets by TargetScan.

Target mRNA	Match Position	Site Type	Site Context Score
Hs-*ADAM10*	490–496	7mer-m8	−0.22
	510–516	7mer-A1	−0.13
Hs-*KLF2*	242–249	8mer	−0.50
Hs-*KLF4*	362–368	7mer-A1	−0.06
	674–681	8mer	−0.41
Mm-*Adam10*	489–495	7mer-m8	−0.22
	509–515	7mer-A1	−0.10
Mm-*Klf2*	214–221	8mer	−0.57
Mm-*Klf4*	433–439	7mer-A1	−0.10
	751–758	8mer	−0.36

## Data Availability

All data included in the manuscript. Supporting information could be requested from the corresponding author.

## Supplementary Materials

The following are available online at https://www.mdpi.com/article/10.3390/biomedicines10010058/s1, Figure S1. (A) qPCR of miR-92a in HCMEC upon normal and high glucose. (B) qPCR of miR-92a expression upon miR-92a inhibition via Ant-92a. (C) qPCR of miR-92a with/without pre-mir-92a overexpression. (D) Western blot of ADAM10 upon overexpression of miR-92a (Pre-92a) compared to siRNA-mediated downregulation of Adam10 (siAdam10) in HCMEC.
