# Supplementary material for: Micro-RNA 92a as a Therapeutic Target for Cardiac Microvascular Dysfunction in Diabetes
Source: Biomedicines. 2021 Dec 28;10(1):58. doi: 10.3390/biomedicines10010058 (PMC8773250; doi:10.3390/biomedicines10010058)
Supplement: Supplementary file 1 [file biomedicines-10-00058-s001.zip › biomedicines-1499586-supplementary.pdf]

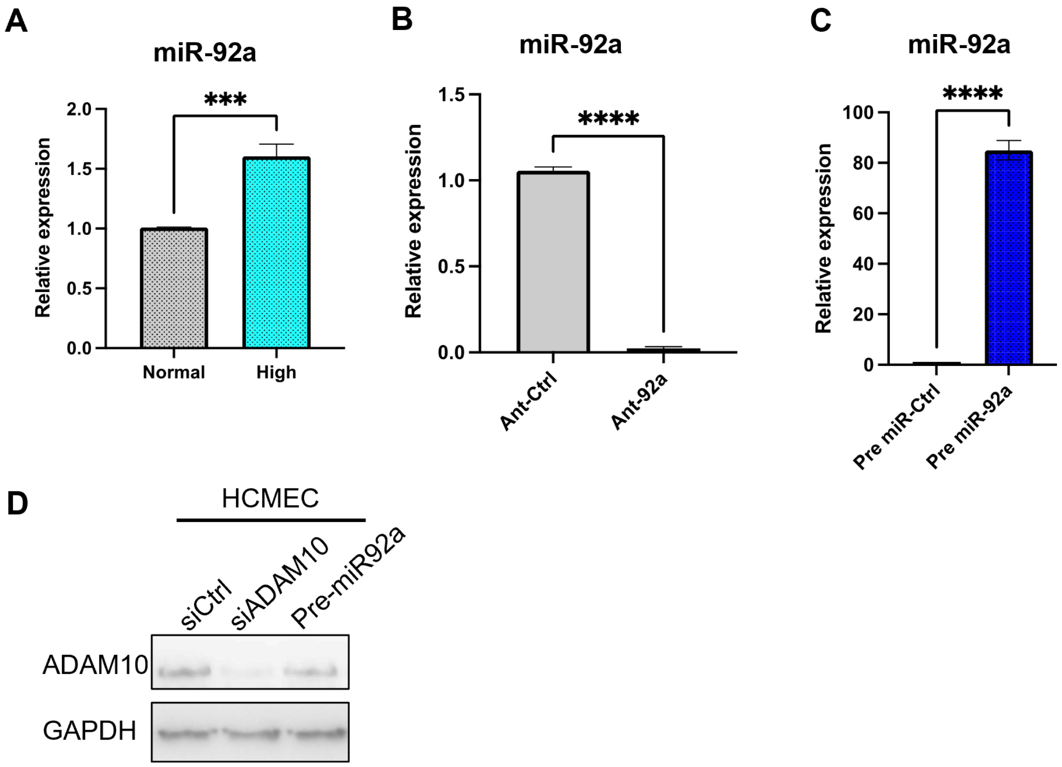

Figure S1. (**A**) qPCR of miR-92a in HCMEC upon normal and high glucose. (**B**) qPCR of miR-92a expression upon miR-92a inhibition via Ant-92a. (**C**) qPCR of miR-92a with/without pre-mir-92a overexpression. (**D**) Western blot of ADAM10 upon overexpression of miR-92a (Pre-92a) compared to siRNA-mediated downregulation of Adam10 (siAdam10) in HCMEC. Statistical analyses by Student's t-test or one-way ANOVA ($N \geq 4$). *** $P < 0.001$, **** $P < 0.0001$.
